# Adjustments to the reference dataset design improve cell type label transfer

**DOI:** 10.3389/fbinf.2023.1150099

**Published:** 2023-04-05

**Authors:** Carla Mölbert, Laleh Haghverdi

**Affiliations:** ^1^ Max Delbrück Center for Molecular Medicine in the Helmholtz Association, Berlin Institute for Medical Systems Biology, Berlin, Germany; ^2^ Department of Biology, Humboldt-Universität zu Berlin, Berlin, Germany

**Keywords:** single-cell RNA-seq, cell type annotation, label transfer, reference data, benchmark, interpretability

## Abstract

The transfer of cell type labels from pre-annotated (reference) to newly collected data is an important task in single-cell data analysis. As the number of publicly available annotated datasets which can be used as reference, as well as the number of computational methods for cell type label transfer are constantly growing, rationals to understand and decide which reference design and which method to use for a particular query dataset are needed. Using detailed data visualisations and interpretable statistical assessments, we benchmark a set of popular cell type annotation methods, test their performance on different cell types and study the effects of the design of reference data (e.g., cell sampling criteria, inclusion of multiple datasets in one reference, gene set selection) on the reliability of predictions. Our results highlight the need for further improvements in label transfer methods, as well as preparation of high-quality pre-annotated reference data of adequate sampling from all cell types of interest, for more reliable annotation of new datasets.

## 1 Introduction

Identification of cell types is an essential part of the analysis of single-cell RNA-seq data, and provides thorough summarizing of the data in light of already existing biological context for the known cell types. Yet, often this is not a straight-forward part of processing and careful cell type annotation is a time consuming process. Recently, more attention has been devoted to the development of methods for transfer of cell type labels from previously annotated datasets to newly acquired data. Several label transfer approaches have been proposed, based on different models such as correlation between the cell states [e.g., Seurat (Stuart et al., 2019), SingleR (Aran et al., 2019), CellID (Cortal et al., 2021)], random forest [e.g., SingleCellNet (Tan and Cahan, 2019)], or deep learning [e.g., ItClust (Hu et al., 2020), SignacX (Chamberlain et al., 2021)]. Existing methods often perform well in predicting cell types of distinct clusters, while cell types without a clear boundary between them (in continuous developmental trajectories, closely related immune cell types, etc.) are more difficult to identify. Reliable annotation of rare cell types is also an important challenge and implies that the prediction quality per cell type needs to be assessed rather than reporting only overall statistics which miss any indication on where (and why) the prediction errors take place.

Here, we use reference and query scRNA-seq datasets from Peripheral Blood Mononuclear Cells (PBMC) samples (Ding et al., 2020) to benchmark five popular label transfer methods and show that the design of the reference dataset should be adapted to the learning approach used by the method. We use reference and query datasets which are complete in respect to each other, meaning that all the cell types in the query are present in the reference and *vice versa*. First, we examine the effects of reference data sampling (i.e., number of cells per cell type) on each method. Using a balance training set is standard in the machine learning field (Kotsiantis et al., 2005) but has mostly been neglected for cell type annotation. Consequently, we implement a weighed bootstrapping-based approach to make use of as much of the reference data as possible, while still keeping the benefits of working with reference data subsets in which cell types are not under-represented. A bootstrapping strategy to deal with low sample numbers in the reference data has been previously used in SignacX (Chamberlain et al., 2021) as well, which offers an ensemble neural networks model pre-trained on the Human Primary Cell Atlas (HPCA) (Mabbott et al., 2013) (for classification of immune cell types). We further show the effect of using reference data from various sources on the different methods, and that a careful selection of the gene set is crucial for the quality of label predictions for high-dimensional and noisy scRNA-seq data. Moreover, we closely examine the implications of confidence scores provided by the different methods and demonstrate that high confidence scores do not directly correlate with correct predictions.

## 2 Results

### 2.1 Less abundant as well as closely related cell types are more difficult to predict

**FIGURE 1:**
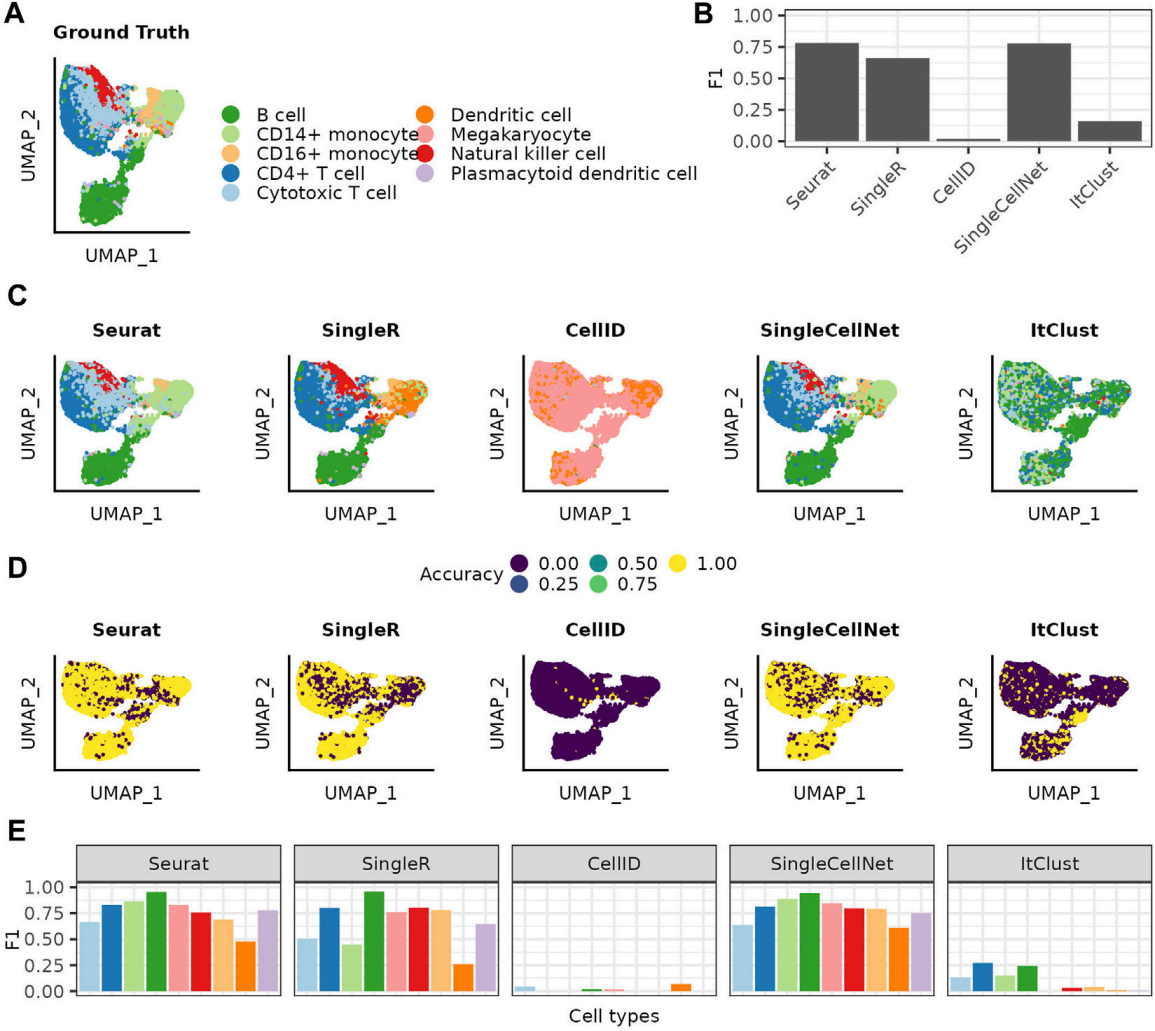
Cell type label transfer on the PBMC dataset using the full reference dataset. **(A)** UMAP of the query cells colored based on the cell types assigned in the ground truth. **(B)** Over all F1 scores achieved for each of the methods. **(C)** UMAPs colored by the cell type annotation made with the different annotation methods. **(D)** UMAPs showing by if a cell was correct or incorrect predicted. **(E)** F1 scores achieved for the different cell types with each of the different methods. The cell types are listed in decreasing order of how often they are represented in the full reference data.

We evaluate the methods on a PBMC dataset, with manually curated cell type labels (Figure 1A). We start by using the entire reference data without any adjustments. Comparing each method’s predictions to the ground truth, we get similar F1 scores for Seurat, SingleR and SingleCellNet and significantly worse performances for CellID and ItClust (Figure 1B). Comparing the ground truth with the annotation from each method, we see that the predictions vary between the different methods, even for methods with similar performances (Figures 1C, D). In general the mispredictions are located in areas of the Uniform Manifold Approximation and Projection (UMAP) (McInnes et al., 2018) where cell types overlap. Taking for example, the area where the two rare cell types of Dendritic cells and Megakaryocytes overlap in the ground truth, we see different predictions with each method. Seurat extends the B cell cluster that lies below, while SingleCellNet predicts a mixture of different cell types in that area. SingleR on the other hand predicts the Dendritic cells in a much wider radius then the ground truth. This shows, that even though these three methods have similar overall F1 scores the prediction for specific populations can vary greatly. This variation is highlighted by the cell type specific accuracy and precision values for each method (Supplementary Figure S1). All methods perform worse for rare cell types as reflected in decrease of F1 scores (Figure 1E). Accounting for such limitations in prediction of less abundant cell types, ItClust even excludes them from modelling, thus does not predict any cells as either Dendritic cells or Megacaryocytes, thereby completely mispredicting all cells belonging to those populations in the query dataset.

### 2.2 Less abundant cell types benefit from more balanced reference data

**FIGURE 2:**
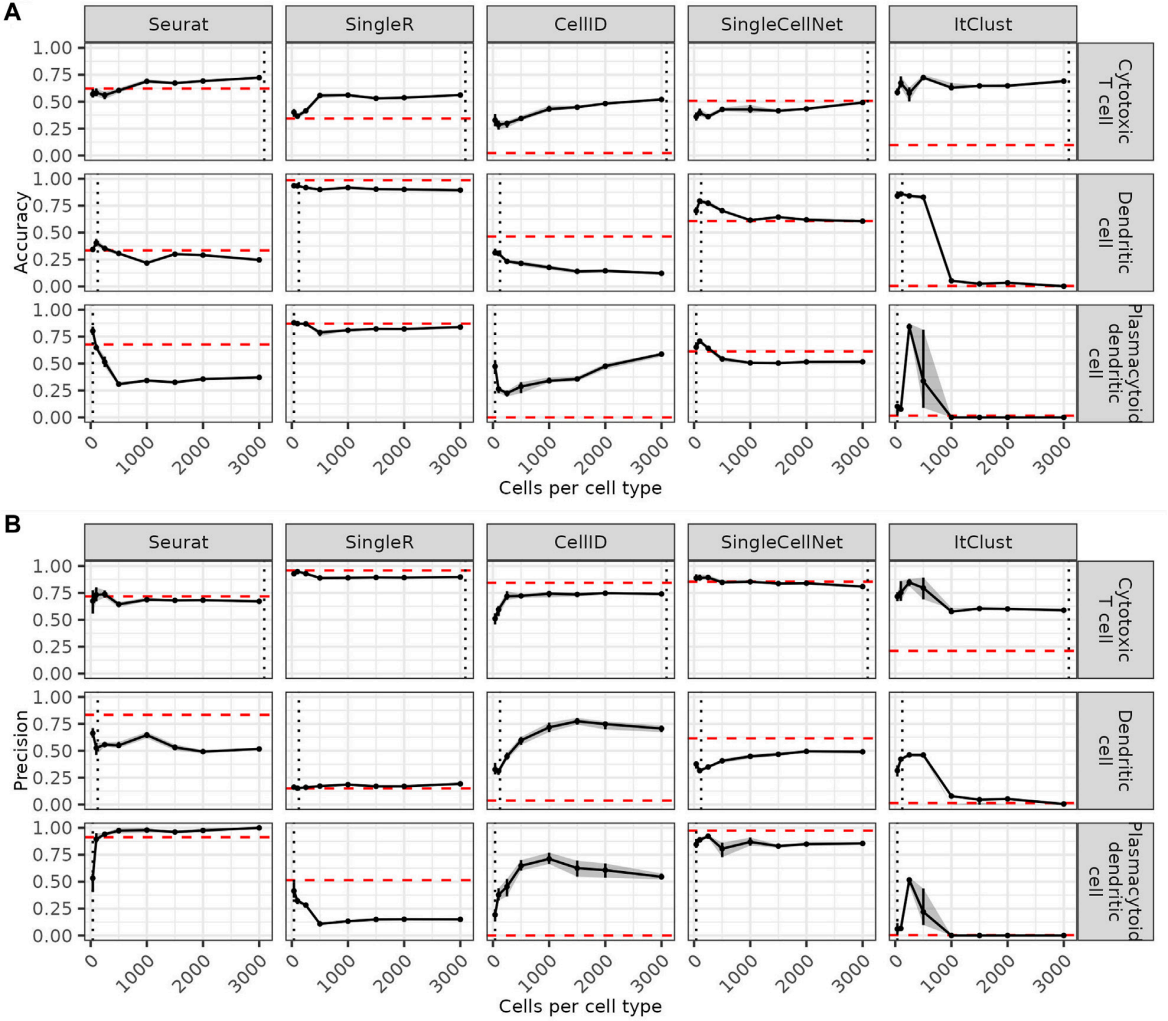
Effect of the number of cells per cell type on the predictions. Accuracy **(A)** and precision **(B)** for three example cell types in each method, when the number of cells per cell type is increased. The red dashed line shows the accuracy on the full data and the grey dashed line shows the number of cells in this cell type in the full reference data. Confidence intervals for 20 bootstraps are shown by a grey shadow.

Since rare cell types are more difficult to predict, we asses how a more balanced reference dataset can affect the predictions. We compare the accuracy of the predictions for each cell type (three example cell types in Figure 2A and the remaining cell types in Supplementary Figure S2) as a function of the maximum number of cells sampled per cell type in the reference dataset. On the one hand, increasing the number of maximum cells per cell type higher than the number of cells available in a cell type leads to a decrease of accuracy for this cell type. This impairment is especially drastic for ItClust where smaller cell types start to be missed completely. CellID is the only method where increasing the number of cells per cell type to more than 1,000 leads to visible improvements. For the other methods even cell types where more data is available are not better predicted after increasing the reference data size beyond a certain threshold. This indicates that limiting the number of cells per cell type to a maximum of 1,000 cells is generally beneficial. On the other hand, including too few cells per cell type has negative effects on the predictions, especially for abundant cell types. When comparing the precision of the predictions as a function of the maximum number of cells per cell type (three example cell types in Figure 2B and the remaining cell types in Supplementary Figure S3) we see that higher accuracy usually comes at the cost of lower precision for each cell type. However, overall the quality of the predictions for each cell type as summarize by F1 scores (Supplementary Figure S4), depends on how well they are represented in the reference data. As a rule of thumb, each cell type tends to be predicted best when the maximum number of cells per cell type is closest to the number of cells available for this cell type. At this point the cell type is best represented without being overshadowed by other more abundant cell types.

### 2.3 Weighted bootstrapping increases the accuracy in the prediction of less abundant cell types

**FIGURE 3:**
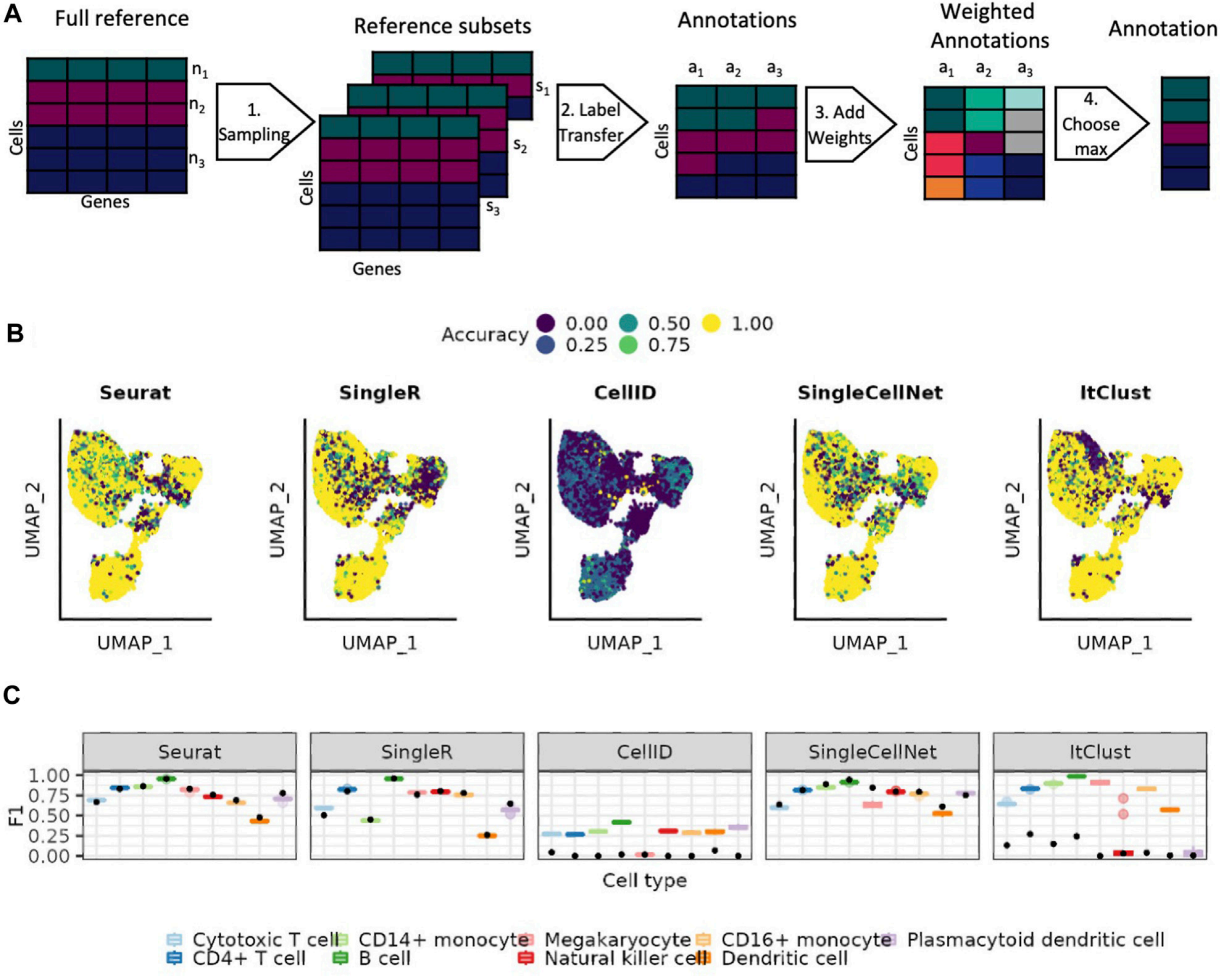
Using weighted bootstrapping for the predictions. **(A)** Overview of the weighted bootstrapping approach. (1) Sampling of the reference dataset with each subset responding to the size of one of the cell types present in the full reference. (2) Cell type label transfer with one of the existing approaches. (3) Adding weights to the annotations based on how well represented the cell type is in the corresponding reference set. (4) Predicting the cell type for which the sum of predictions is the highest. **(B)** UMAP colored by the difference between the mean accuracy achieved when using the bootstrapping-based approach *versus* the one achieved on the individual reference subsets. **(C)** Distribution of the F1 scorers for the individual reference subsets shown as boxplots (see Methods). The black points represents the result achieved on the full reference set.

We implement a weighted bootstrapping-based approach, that allows us to account for the variable abundances of different cell types in the reference data (Methods 4.4 and Figure 3A). We select subsets where the maximum number of cells per cell type align with the abundance of one of the cell types in the reference. The predictions made on each of these subsets is then weighted based on how close the abundance of the predicted cell type is to the maximum number of cells in this subset. This approach allows us to use as much of the data as possible, while still weighting the prediction of cell types higher when they are better represented in the reference data subset. Figure 3B shows how well each cell is predicted across 20 bootstrapping sets. We observe that the most uncertain prediction areas by bootstrapping generally mirror the results of using the full data shown Figure 1D. With bootstrapping, the absolute value of accuracy increases for all smaller cell types but their precision tends to decrease (Supplementary Figure S5), especially visible for Seurat and SingleCellNet. Nevertheless, the F1 scores indicate that the benefits in the accuracy outweigh the difficulties in the precision (Figure 3C). SingleR is not much affected by the bootstrapping, neither positive nor negative. ItClust and CellID perform better for all cell types with bootstrapping. ItClust no longer misses any of the cell types completely.

**FIGURE 4:**
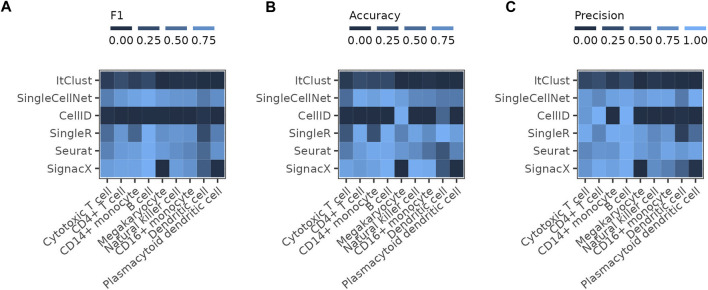
Comparing SignacX to the other methods The figure shows the **(A)** F1 score, **(B)** accuracy and **(C)** precision of the individual methods reached on the full PBMC reference dataset compared to the results achieved by SignacX on the pre-trained model for immune cell type prediction on the Human Primary Cell Atlas (HPCA) reference dataset.

We further assess the performance of the pre-trained SignacX immune cell types labeling tool on the PBMC data, which uses bootstrapping on the HPCA data as the reference for the training of the model. The performance of SignacX is comparable to SingleR, Seurat and SingleCellNet for most cell types (Figure 4). The reference data of SignacX does not contain any Megakaryocytes and therefore misses predicting them completely.

### 2.4 Including data from multiple sources allows more balanced coverage of all cell types

**FIGURE 5:**
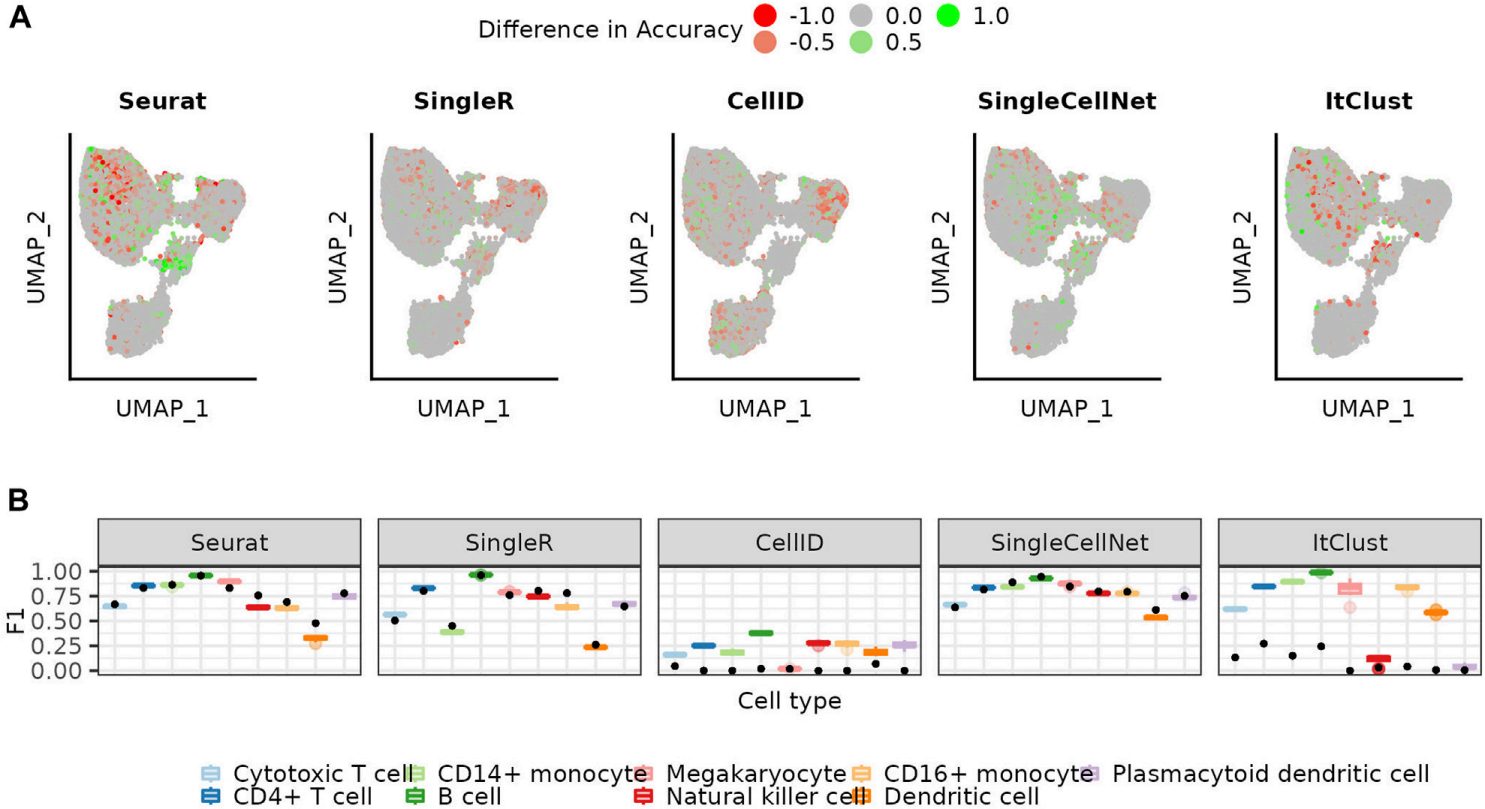
Using mosaic reference data *versus* the mono-source reference. **(A)** UMAP colored by the mean accuracy achieved on the mosaic references subtracted by the mean accuracy achieved on the mono-source references when using the weighted bootstrapping approach **(B)** Distribution of the F1 scores for the mosaic (colored boxplots, see Methods) for the weighted bootstrapping-based approach. The initial performance on the full reference is shown as a comparison (black dot).

With the increasing number of annotated datasets, it becomes possible to combine multiple existing datasets into the reference. To simulate this we extended our reference with cells from other sequencing technologies and repeated the annotation using the bootstrapping approach. The UMAPs colored by the difference in accuracy for each cell when using a mosaic-reference set compared to a mono-source reference set, show that most cells have a similar performance for both sets and that changes mostly occur in difficult areas for each method (Figure 5A). Accuracies for Seurat increase in some areas while decreasing in others, while SingleCellNet accuracies mostly increase by using mosaic reference data. Precision values also differ only slightly between mono-source and mosaic reference data (Supplementary Figure S6). Taking a look at the cell type specific F1 scores (Figure 5B), we conclude the use of mosaic data does not introduce significant batch effect artifacts to the predictions, thus can be helpful for more balanced representation for all cell types in the reference set.

### 2.5 Selection of the gene set affects the methods differently

**FIGURE 6:**
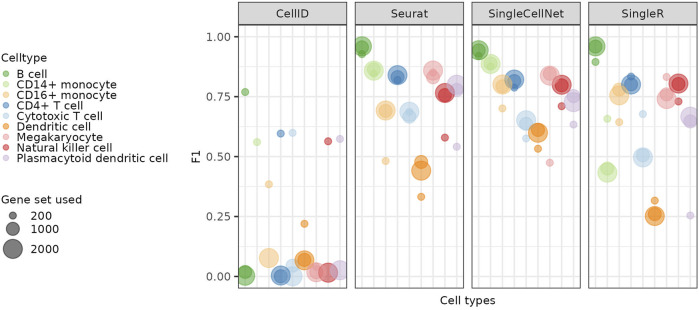
Comparing predictions based on different gene sets Cell type specific F1 scores achieved with the different gene sets for each of the methods. The 1,000 and 2000 genes sets yield better predictions than the 200 genes set for most methods and cell types.

In this section, we show that the selection of genes included in the data also affects the learning models and thus the label transfer process. In the previous sections we used a set of 1,000 highly variable genes (HVGs) for the predictions, as most single-cell RNA-seq analysis pipelines use roughly a similar number of top HVGs. To test whether using a different amount of genes would affect the predictions by the different methods, we reran all the methods with 200 and 2000 HVGs. In Figure 6 we show the F1 scores achieved when using 200, 1,000 or 2000 HVGs. While the gene set affects the performance, the results differ significantly between the different methods. Seurat and SingleCellNet (which have their own inner procedures of feature selection, i.e., by data compression and random forest respectively, see Methods) perform less well when using 200 HVGs but increasing the number of HVGs from 1,000 to 2000 does not lead to further improvements. For SingleR the abundant cell types benefit from a low number of HVGs, while the less abundant cell types are better predicted with more HVGs. CellID shows the biggest difference in accuracy and prediction depending on HVG selection and performs significantly better with 200 HVGs. The improvement for CellID is mostly in accuracy, except for the Dendritic cells which get better in the precision (Supplementary Figure S7). The Megakaryocytes remain over-predicted independent of the number of genes. ItClust includes internal (hard-coded) filtering of the gene set, and was therefore excluded for the analysis in this section.

As an additional test, we use the Triana et al. (2021) bone marrow dataset with 560 manually curated genes (with known biological relevance) to annotate a second healthy human bone marrow dataset sequenced in the same study, using the weighted bootstrapping approach (Supplementary Figure S8). ItClust benefits significantly from the bootstrapping for all cell types and SingleCellNet performs better for rare cell types. However, other methods show no improvements using the bootstrapping approach and perform even less well for some cell types. We anticipate this happens because the curated set of genes reliefs the model training for these methods, therefore the less abundant cell types are adequately modeled even without bootstrapping This indicates that the careful selection of the gene set can compensate for an unbalanced reference dataset.

### 2.6 High confidence scores do not always align with correct predictions

**FIGURE 7:**
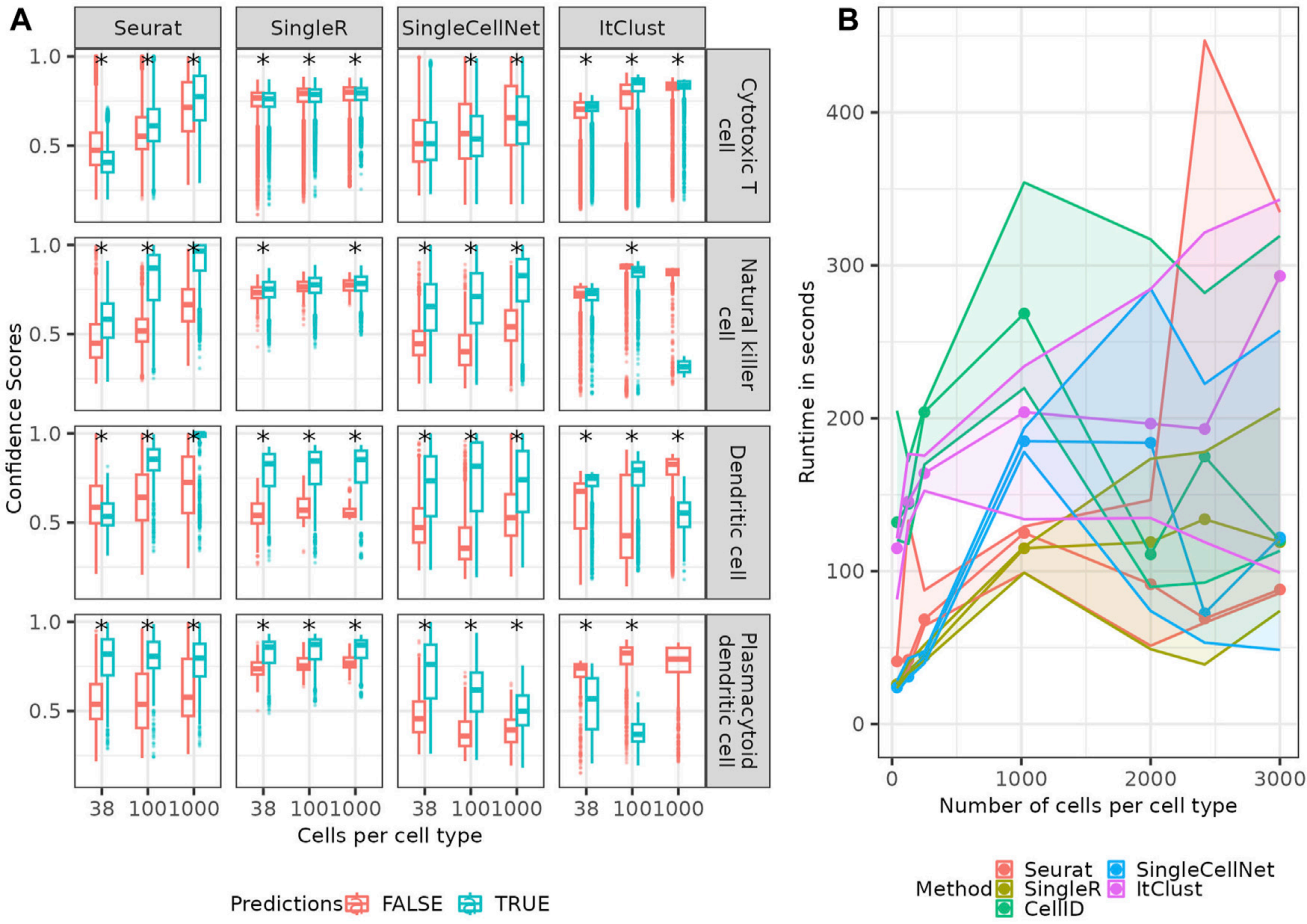
Confidence scores and runtime. **(A)** Boxplots (see Methods) of confidence-scores for the predicted cell types depending of true and false predictions of each of the methods providing confidence scores for four example cell types. The star signs indicate significant (*p*-value < 0.05, see Methods) difference between the False and True predictions. **(B)** runtime in seconds on a CentOS7 cluster with a memory limit of 16G on one core with 8CPU available.

Four of the five methods of interest supply a confidence score for the predicted cell type. In Figure 7A; Supplementary Figure S9 we show the confidence scores of each the method separated in true and false predictions. While Seurat shows a clear difference in distribution of confidence scores, for true and false predictions for many cell types, there are cell types where this difference is rather small, such as the Cytotoxic T cells and the natural killer cells. For less abundant cell types such as the Dendritic cells and the Plasmacytoid dendritic cells, the true predictions have generally a higher confidence than the false predictions, for smaller numbers of cells per cell type. But with an increase in the number of cells, Seurat starts to become even more confident in the false predictions, making the confidence scores less helpful. This implies, the method does not model the rare cell types correctly and only becomes more confident in a false model for them when provided larger number of cells which make the data more imbalanced. The confidence scores supplied by SingleR show significantly higher confidence for correct predictions in the rare cell types (non-overlapping error bars for correct and false predictions indicate a significant difference between them), but no clear difference between the two distributions for other cell types e.g., Cytotoxic T cells. This implies a good modeling of rare cell types, but again not a good model for cell types with mixing boundaries. Similarly, for SingleCellNet the confidence in the correct predictions is generally (but not for all cell types) higher than the one in false predictions, which is good. For ItClust, the confidence scores appear as rather random and uninformative. The (partial) discordance we observe between confidence scores and correctness of predictions across the methods is not surprising, as the confidence score reflects the variation in prediction for a query cell given a model; when the model is wrong (or not good enough), it can make wrong predictions quite confidently. CellID does not provide confidence scores, so it was excluded for the analysis in this section.

### 2.7 Reporting the runtimes

In addition to the prediction quality of the different methods we checked their runtime (Figure 7B), which revealed that the runtime of all methods increases linearly or slower with the number of cells in the reference. In all trials the number of input genes (1,000) as well as the query data was fixed. Seurat and SingleR are the two most computationally efficient methods.

## 3 Discussion

In this work we benchmarked five popular label transfer methods on PBMC datasets and showed how the selection and treatment of the reference data affects the quality of the prediction. We demonstrated the effects of data sampling in the reference and showed that all methods tend to predict highly represented cell types better than rare cell types. We found that reducing the reference data to balance the cell types improves the ability to predict less abundant cell types. However, disregarding too many cells to match the number of available cells for every cell type results in lower accuracy of abundant cell types. Overall methods that include an explicit modelling step (different kinds of data compression, deep learning, etc.) benefit from more balanced reference sets, in contrast to methods that rely only on cell states’ correlations such as SingleR. To make a sensible compromise between data balancedness and utilization, we implemented a weighted bootstrapping method that includes the predictions of multiple different sized and more balanced subsets. This approach generally improves the predictions for ItClust and CellID. The other methods do not show a change in the F1 scores, but the accuracy of predicting the rare cell types increases. This indicates that depending on the research question and the importance of finding less represented cell types, more balanced reference sets and the weighted bootstrapping can be beneficial for these methods as well. Prior to our study, bootstrapping to enhance the statistical power for prediction of rare cell types has also been used in SignacX. While their bootstrapping approach was implemented for training their (neural network) model on a specific bulk reference dataset, we show that bootstrapping can be beneficial for other label transfer methods and model settings, as well.

As the number of available annotated datasets is growing, one can consider combining multiple datasets as a potential for increasing the statistical power for making more accurate predictions, especially for less abundant cell types. However, other factors such as technical differences and batch effects between the datasets could introduce new causes for mispredictions. We combined datasets from multiple sources (without batch correction) as one reference and evaluated how this affected the labels transfer. We found in general, mosaic data does not weaken the overall performance for any method. Thus, especially in cases where combining multiple reference datasets would allow identification of additional cell types, we would suggest to do so. Also in rare cell types, such as the Plasmacytoid dendritic cells for which the minimum number of cells is increased from 38 in the mono-source to 102 in the mosaic data, additional data appears beneficial.

When determining distances between different cell states in high-dimensional space, we often face the challenge of the curse of dimensionality (Imoto et al., 2022). The higher the number of dimensions, the more severe the issue, as noise disproportionately adds up to undermine the true (biological) signal when considering multiple dimensions. Because of this, one could expect that reducing the number of dimensions helps with better defining cell similarities (and distances), thus improving the predictions. Previous studies (Schraivogel et al., 2020; Triana et al., 2021) have indicated that a curated set of genes with known biological relevance facilitates reliable cell type label identification for the bone marrow tissue, even at very low reads depth. However, such prior knowledge may not be at hand for the data analyst. Without using prior knowledge, we compared performances between gene sets of different sizes on the PBMC data and showed that the number of included HVGs alone is not determinant of the predictions quality. In fact using different gene sets affected different cell types and methods differently.

Furthermore, we demonstrated that confidence scores provided by different methods, cannot be taken as an absolute measure for correctness of predictions, but rather they show the robustness of prediction for a query cell assuming the model used by the method is correct. Correlation between the confidence scores and correct predictions (when ground truth labels are available) would indicate the reliability of a model. Overall, SingleR showed one of the most robust and reliable performances in our benchmarking experiments using PBMC reference and query datasets that were complete with respect to each other. Seurat and SingleCellNet’s performances were also generally good and competing with SingleR, but we assessed CellID and ItClust as not being very robust and reliable. However, one could expect the performances to be different for other data scenarios, which thus need to be further tested, e.g., scenarios in which one or a few cell types are exclusively present in either the query or the reference set, or when much bigger reference data is available. In our study, none of the available methods were able to make highly accurate predictions for all cell types even with careful design of the reference. In all cases at least one of the cell types has an accuracy below 0.5 even if other cell types reach accuracy values close to 1. This could be due to inadequate performance of the methods, but also to incorrectly labeled cells in the reference and query data that we assume as ground truth. The annotations assumed as ground truth in this study have been attained in the original publication Ding et al. (2020) using standard single-cells data clustering and annotation algorithms, which may include errors. As Figure 1 indicates, annotation errors tend to happen more along the boundaries between closely related cell types (e.g., in continual developmental trajectories). The definition of these boundaries diverges in different annotations, which makes some degree of error in such regions inevitable.

To conclude, our study highlights the need for further improvement of cell type label transfer methods as well as better reference data quality (i.e., original cell types annotation by clustering, etc.) and design acquisition, as two major bottlenecks that require simultaneous attention and refinement for improving state-of-the-art reliability of labels transferred to new data. With restriction to the existing methods and data, we showed that predictions of different methods can be improved by a careful design and assembly of the reference dataset. In particular, this design needs to be adapted to the method used.

## 4 Materials and methods

### 4.1 Data and code availability

We performed our analysis on publicly available human PBMC cell (Ding et al., 2020) data (Table 1, Gene Expression Omnibus accession number: GSE132044). The data has been annotated in the original publication, which we use as the ground truth cell labels. This annotation was done based on assigning cell type labels to non-overlapping clusters created using the Louvain community detection algorithm on the scRNA-seq dataset. Manually curated marker genes for each cell type were used to assign a cell type to each cluster. A more detailed description of the annotation process and the list of marker genes can be found in the original publication of the data.

**TABLE 1:** Description of the PBMC datasets included in this study.

Dataset	No. Cells	No. Genes	No. Classes	Protocol
PBMC Query	11,183	33,658	9	Smart-Seq2, CEL-Seq2, 10X, Drop-Seq, Seq-Well, inDrops
PBMC 10X	9,666	33,658	9	10X
PBMC CEL-Seq	253	33,658	7	CEL-Seq2
PBMC Drop-Seq	3,222	33,658	9	Drop-Seq
PBMC inDrops	3,222	33,658	7	inDrops
PBMC Seq-Well	3,176	33,658	7	Seq-Well
PBMC SMART	253	33,658	6	Smart-Seq2

To strengthen our claims we repeat part of the analysis on human bone marrow data published by Triana et al. (2021). In their study they published multiple human bone marrow datasets, here we use the one containing bone marrow from healthy young and old donors (https://doi.org/10.6084/m9.figshare.13397651.v2) as reference and a cells from young healthy donor (https://doi.org/10.6084/m9.figshare.13397987.v3) as query (Table 2). Since the reference dataset was sequenced on a targeted gene set, we will use the 560 curated genes in the cell type label transfer.

**TABLE 2:** Description of bone marrow datasets included in this study.

Dataset	No. cells	No. genes	No. classes	Protocol
BM Query	13,165	560	13	CITEseq
BM Reference	49,057	560	14	CITEseq

The code for reproducing the results and figures in this study is available on GitHub (https://github.com/HaghverdiLab/CelltypeLabelTransfer).

### 4.2 Preprocessing

The PBMC data in (Ding et al., 2020) contains two annotated experiments run on different days. Both experiments contain samples from all scRNA-seq platforms. In Sections 2.1, –Section 2.3 and Section 2.4–Section 2.7 we use exclusively the cells in “experiment one” which were gathered on the 10X platform as our reference data, which we refer to as the mono-source reference. In Section 2.4 we combine the 10X data with all the other scRNA-seq platforms (i.e., Smart-Seq, CEL-Seq2, 10X, Drop-Seq, Seq-Well, inDrops) from the same experiment into one reference, which we refer to as the mosaic reference. We combine the different sets without applying any batch corrections. We use experiment two as query data. The query and the reference data contain the same 9 cell types (Cytotoxic T cell, CD4^+^ T cell, CD14^+^ monocyte, B cell, Megakaryocyte, Natural killer cell, CD16^+^ monocyte, Dendritic cell, Plasmacytoid dendritic cell).

In order to make the different label transfer methods comparable, we use the same data preprocessing workflow for all methods as far as possible. The preprocessing of the gene expression count data starts with selecting the top 1,000 (200 or 2,000 HVGs) using the Pearson residuals method (Lause et al., 2021). We then do a log transformation of the highly variable genes (HVGs) read counts followed by cell-wise L2 normalization. To allow the log transform on the expression matrix, we add a small value (0.001) to the expression values in order to avoid zero values. However, these preprocessing steps could not be used for ItClust, for which the preprocessing steps are hard-coded in the package and cannot be changed. Minor adjustments to the preprocessing workflow to meet each method’s requirements are described in its corresponding Methods section.

### 4.3 Reference data permutation and confidence intervals

To analyze the importance of reference data selection, we assess the different methods, using subsets of the initial reference data of varying size. We vary the maximum numbers of cells per cell type (38, 100, 250, 500, 1,000, 1,500, 2,000, 3,000). We note that if a cell type has less then the maximum number of cell types the reference data will be unbalanced. For each of the reference set sizes, 20 random permutations were sampled from the underlying reference data.

### 4.4 Weighted bootstrap-annotation of cell types

We implement a weighted bootstrap-annotation approach, to assign a weight to the predictions, based on how the predicted label is represented in the full reference. Each query cell *i* gets assigned a label *l*_*i*,*j*_ for each reference set *r*_*j*_:

$$w\left(l_{i,j},r_{j}\right)=\frac{1}{\left(\left|n\left(l_{i,j}\right)-\max\left(r_{j}\right)\right|+1\right)}$$ (1)

Where *n*(*l*_*i*,*j*_) is the number if cells annotated with label *l*_*i*,*j*_ in the full reference data and max(*r*_*j*_) is the maximum number of cells per cell type in reference set *r*_*j*_. For each cell type the different weights are added up and the cell type with the highest weight is predicted. Here, we select a subset for each cell type in the reference data with the maximum number of cells per cell type being the number of cells for this cell type in the full reference.

### 4.5 Specification of the boxplots

In all boxplots in the figures of this manuscript, the box fills the interquartile range (*IQR*)between the 25th (*Q*1) and the 75th (*Q*2) percentile and the line through the box shows the median of the distribution. The lines extending from the box show the minimum (*Q*1 − 1.5*IQR) and maximum (*Q*3 + 1.5*IQR) value in the data. Values outside of this range are plotted as individual points and are considered potential outliers.

### 4.6 Seurat

We follow the Seurat label transfer workflow as suggested in the “Mapping and annotating query datasets” vignette. The Seurat algorithm consists of two steps. The first is an unsupervised compression of the reference and query data into a common space that captures the most correlated features between the two datasets, by using Canonical Correlation Analysis (CCA). This step implies that the query data distribution (as well as the reference data) affects the data compression model which is used in the next step for label transfer. In the second step, the most common label among the set of mutual nearest neighbors (MNN) of a query cell (Haghverdi et al., 2018) in the reference set is transferred to it as the predicted label. We use version v4.2.0 of the Seurat R package.

### 4.7 SingleR

SingleR uses an iterative approach to transfer cell type labels from prior annotated reference data to an unannotated query dataset. The annotation process is performed individually for each query cell. First the variable genes among the cell types in the reference set are selected. Secondly, the Spearman correlation between the query cell and each cell in the reference is calculated. The correlation values are aggregated by cell type and the cell type with the lowest correlation value and all cell types with a correlation more then 0.05 smaller then the top value are removed. The steps are then repeated until only 2 cell types remain and the cell type with the higher correlation is predicted. We use version v2.0.0 of the SingleR R package.

### 4.8 CellID

CellID uses Multiple Correspondence Analysis (MCA) for data compression (unsupervised step) as well as to identify per-cell gene signatures. The signatures of the reference and query data are then compared using a hypergeometric test. The label of the closest cell in the reference is then transferred to the corresponding query cell. Gene set selection is part of the CellID workflow, therefore instead of initially selecting 200 HVGs, we select the top 5,000 HVGs and use CellID’s specific gene set selection method to reduce the number of genes to 200. We note that this gene set varies from the one used by the other methods. We use version v1.6.0 of the CellID R package.

### 4.9 SingleCellNet

SingleCellNet is a random forest based approach, that transforms the data into a cell-by-cell binary matrix derived by pairwise comparison of selected genes (Top-Pair transformation). The workflow starts with reducing each cell type to a fixed number of cells (default: 100 cells). Here, we skip this step. This is followed by training the model. We use the default settings. The labels are transferred to the query data, based on the random forest classifier. SingleCellNet allows to set a number of random profiles to the evaluation process, which allows to identify if cells might belong to a cell type not represented in the reference data. We treat these cells as false predictions. We use version v0.4.1 of the SingleCellNet R package.

### 4.10 ItClust

ItClust is an iterative transfer learning approach for clustering and cell annotation. The neural network model is trained in two steps. It starts with supervised learning on the reference data followed by an additional learning step on the query data to fine-tune the parameters. We use ItClust with its default settings, which includes a gene set selection and a filtering of reference and query cells. We treat removed query cells as false predictions. Since ItClust includes its own preprocessing steps, we did not apply our preprocessing workflow. We use version v1.2.0 of the ItClust python package.

### 4.11 SignacX

SignacX is an R package of a neural network model trained on bulk flow-sorted RNA-seq data of immune cell types from the Human Primary Cell Atlas (HPCA) (Mabbott et al., 2013). To deal with low sample numbers for some of the cell types, SignacX performs a bootstrapping on the HPCA reference data to train an ensemble of *n* = 100 neural network classifiers. The pre-trained SignacX model has been shown to be able to identify immune cell types across a variety of diseases, tissues and sequencing technologies (Chamberlain et al., 2021).

## Data Availability

The original contributions presented in the study are included in the article/Supplementary Material, further inquiries can be directed to the corresponding author.
