# Supplementary material for: Adjustments to the reference dataset design improve cell type label transfer
Source: Front Bioinform. 2023 Apr 5;3:1150099. doi: 10.3389/fbinf.2023.1150099 (PMC10114588; doi:10.3389/fbinf.2023.1150099)
Supplement: Supplementary file 1 [file DataSheet1.PDF]

# Supplementary Material

## 1 FIGURES

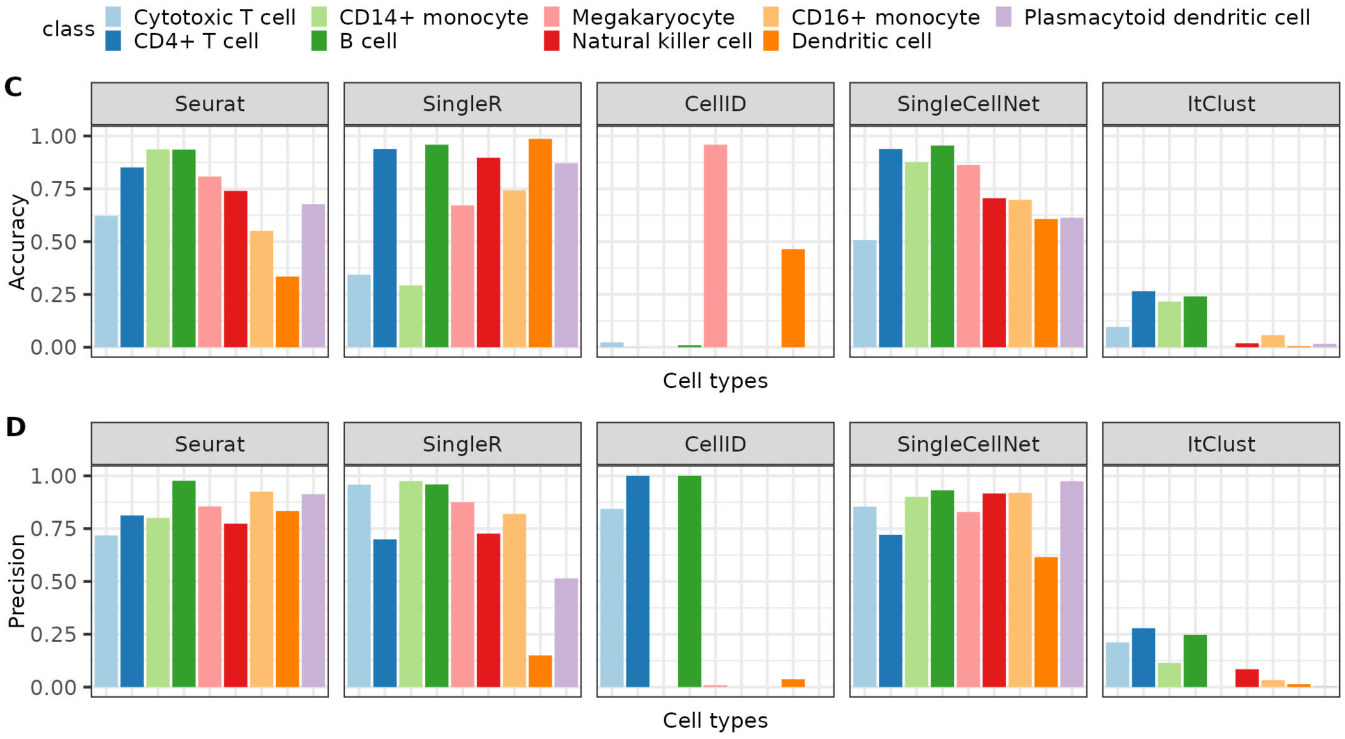


**Figure S1. Cell type label transfer on the PBMC dataset using the full reference dataset.** (A) Accuracy and (B) Precision achieved for the different cell types with each of the different methods. The cell types are listed in decreasing order of how often they are represented in the full reference data.

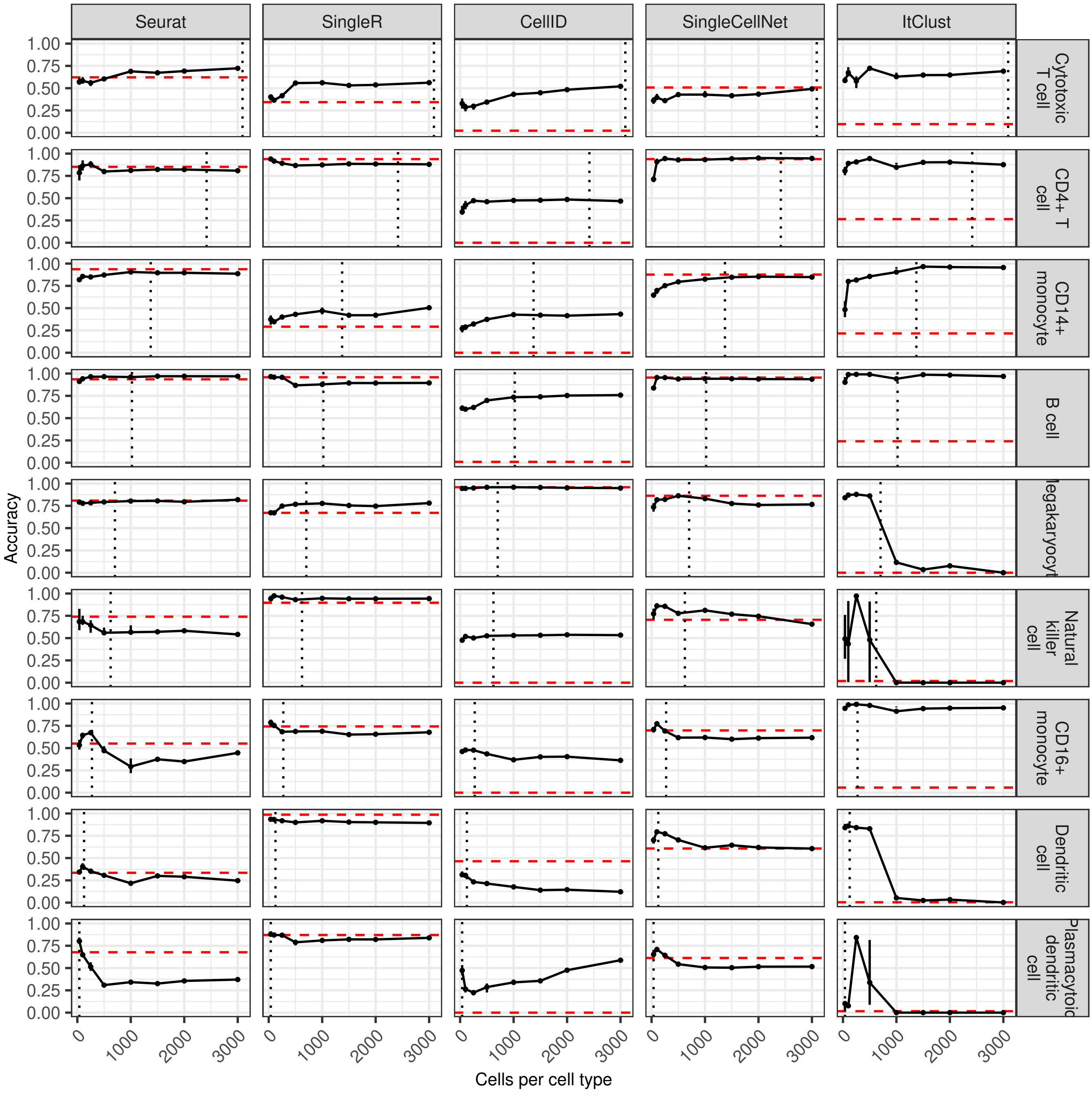


**Figure S2. Effect of the reference data composition on the accuracy of the predictions.** Distribution of the accuracy for each cell type for each method, when the number of cells per cell type is increased. The red line shows the accuracy on the full data and the grey line shows the number of cells in this cell type in the full reference data.

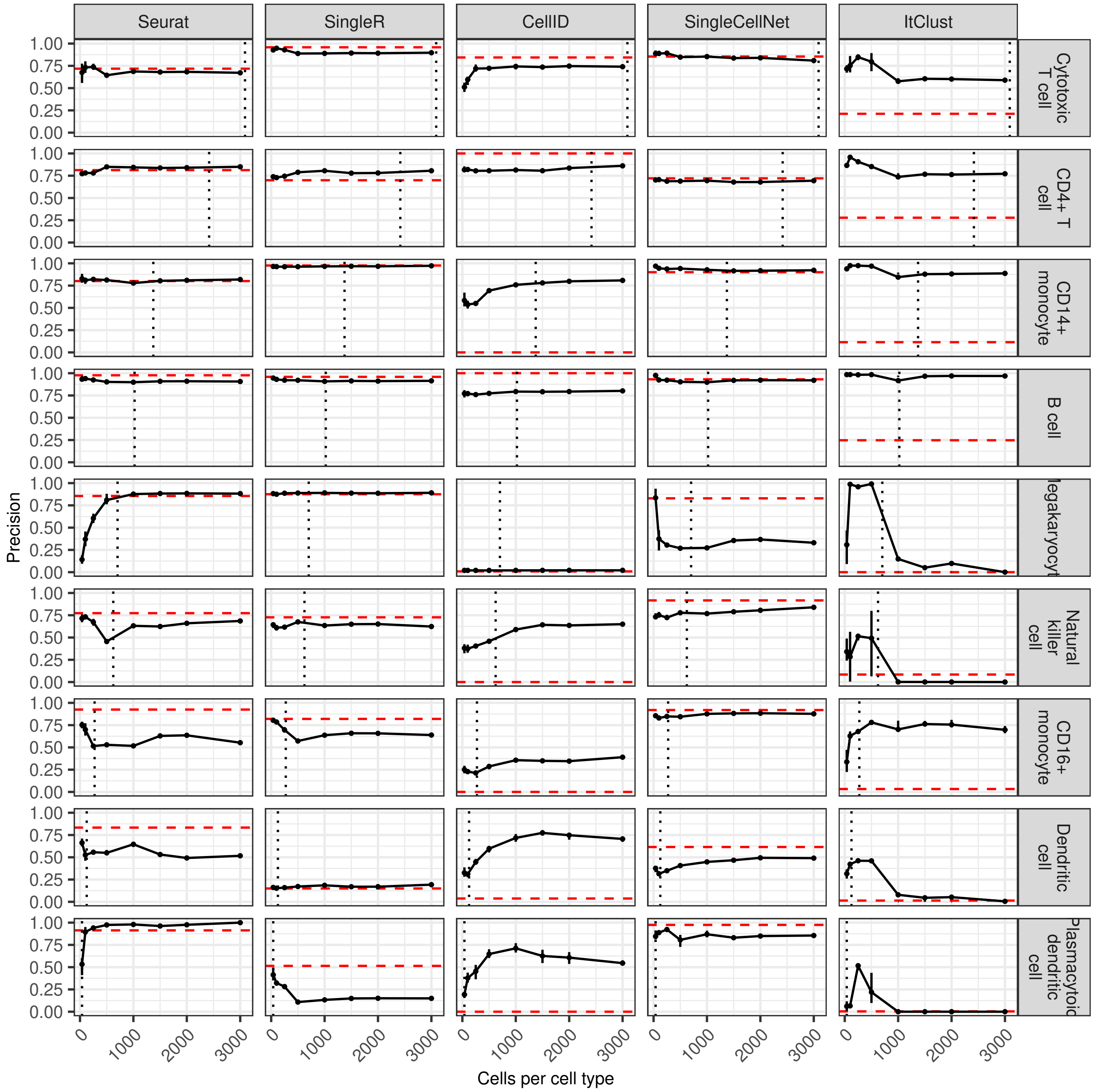


**Figure S3. Effect of the reference data composition on the precision of the predictions.** Distribution of the precision for each cell type for each method, when the number of cells per cell type is increased. The red line shows the accuracy on the full data and the grey line shows the number of cells in this cell type in the full reference data.

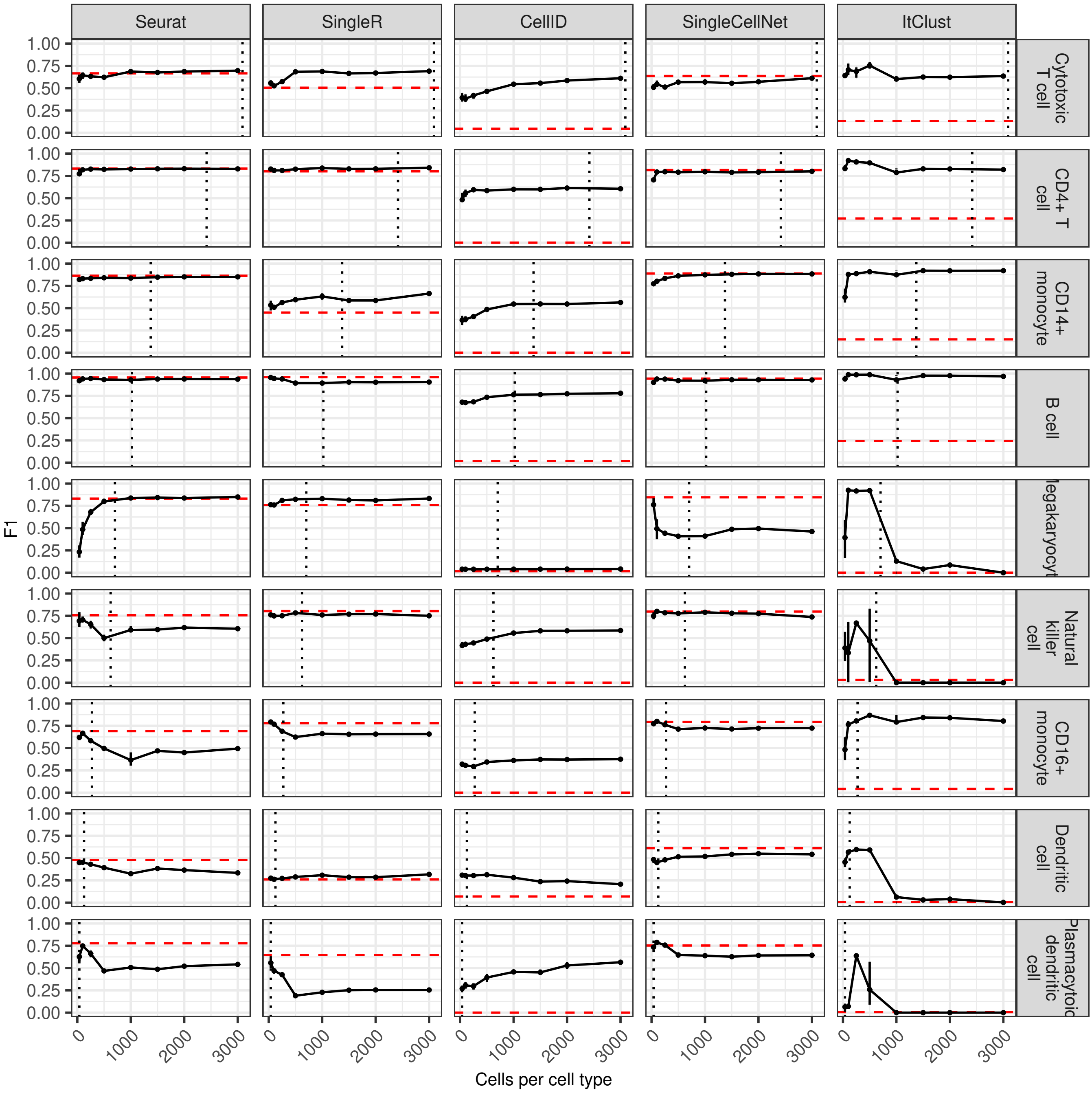


**Figure S4. Effect of the reference data composition on the F1 scores achieved for the predictions.** Distribution of the F1 for each cell type for each method, when the number of cells per cell type is increased. The red line shows the accuracy on the full data and the grey line shows the number of cells in this cell type in the full reference data.

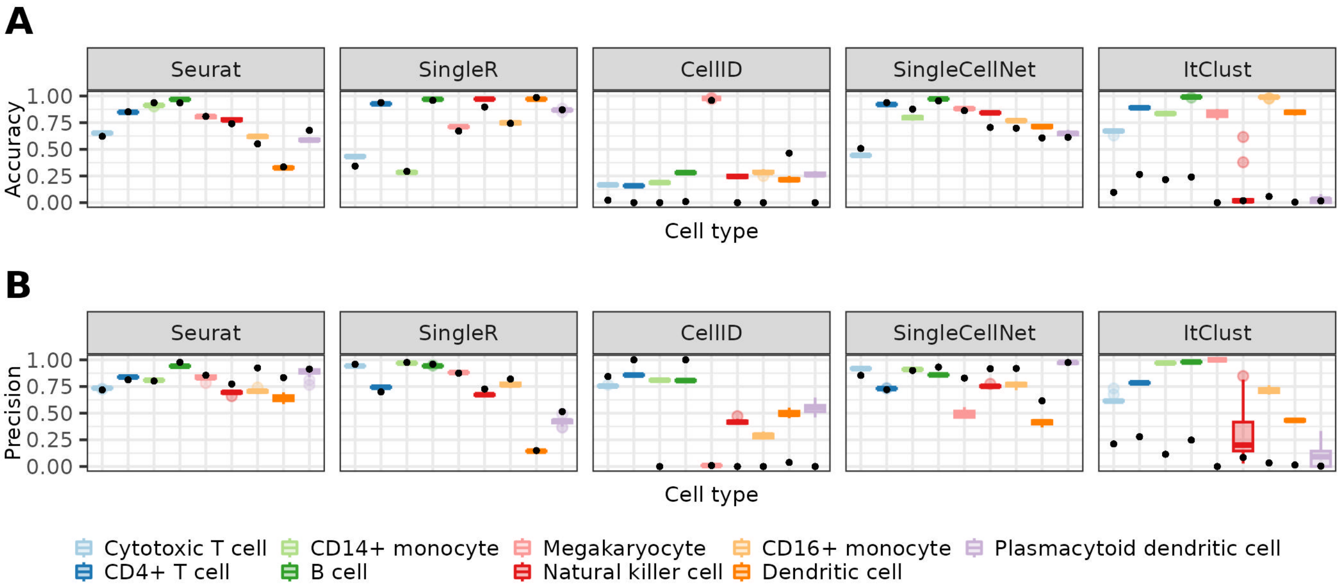


**Figure S5. Combining existing label transfer methods with weighted-bootstrapping on mono-source reference data.** (A) accuracy and (B) precision for each cell type for each method, when applying the methods in combination with weighted bootstrapping. The black point shows how the performance achieved with the full reference data.

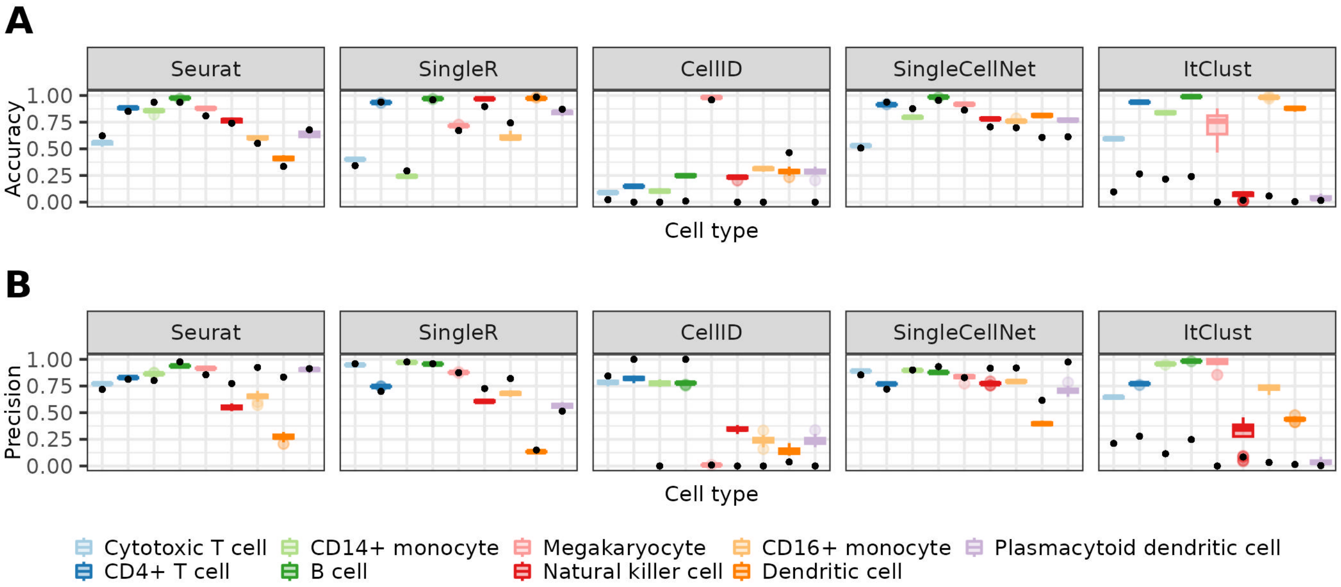


**Figure S6. Combining existing label transfer methods with weighted-bootstrapping on mosaic reference data.** (A) accuracy and (B) precision for each cell type for each method, when applying the methods in combination with weighted bootstrapping. The black point shows how the performance achieved with the full reference data.

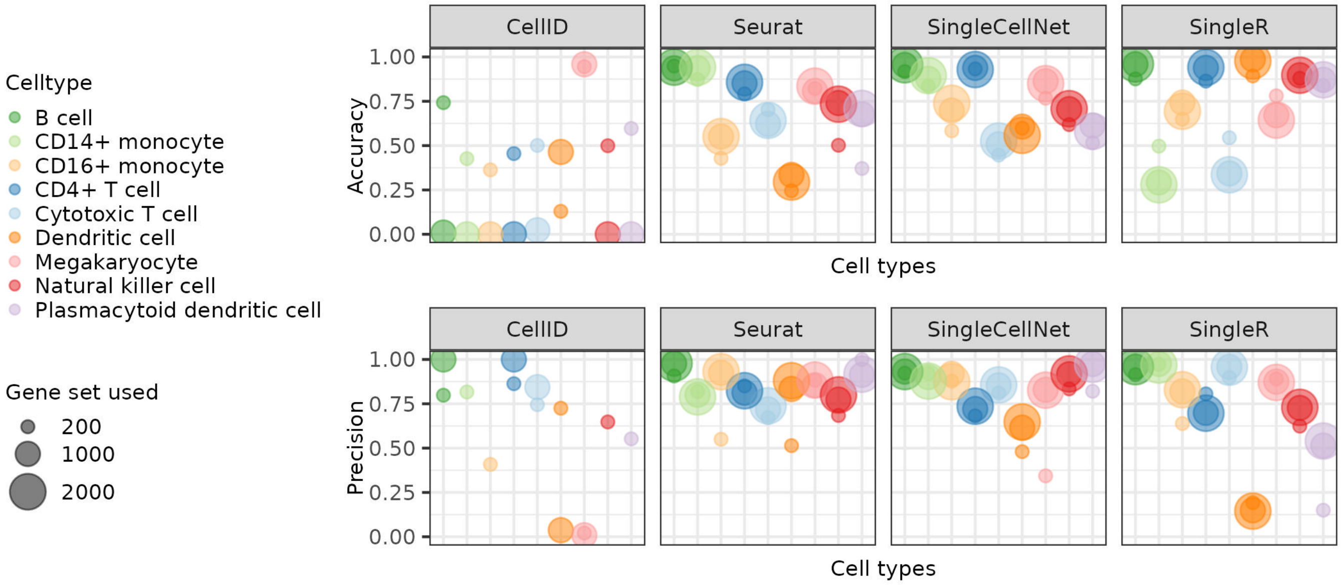


**Figure S7. Effects of the number of HVGs selected.** (A) Accuracy and (B) Precision values achieved on the individual cell tyoes with gene sets of different sizes (200, 1000, 2000) on the full mono-source PBMC reference dataset.

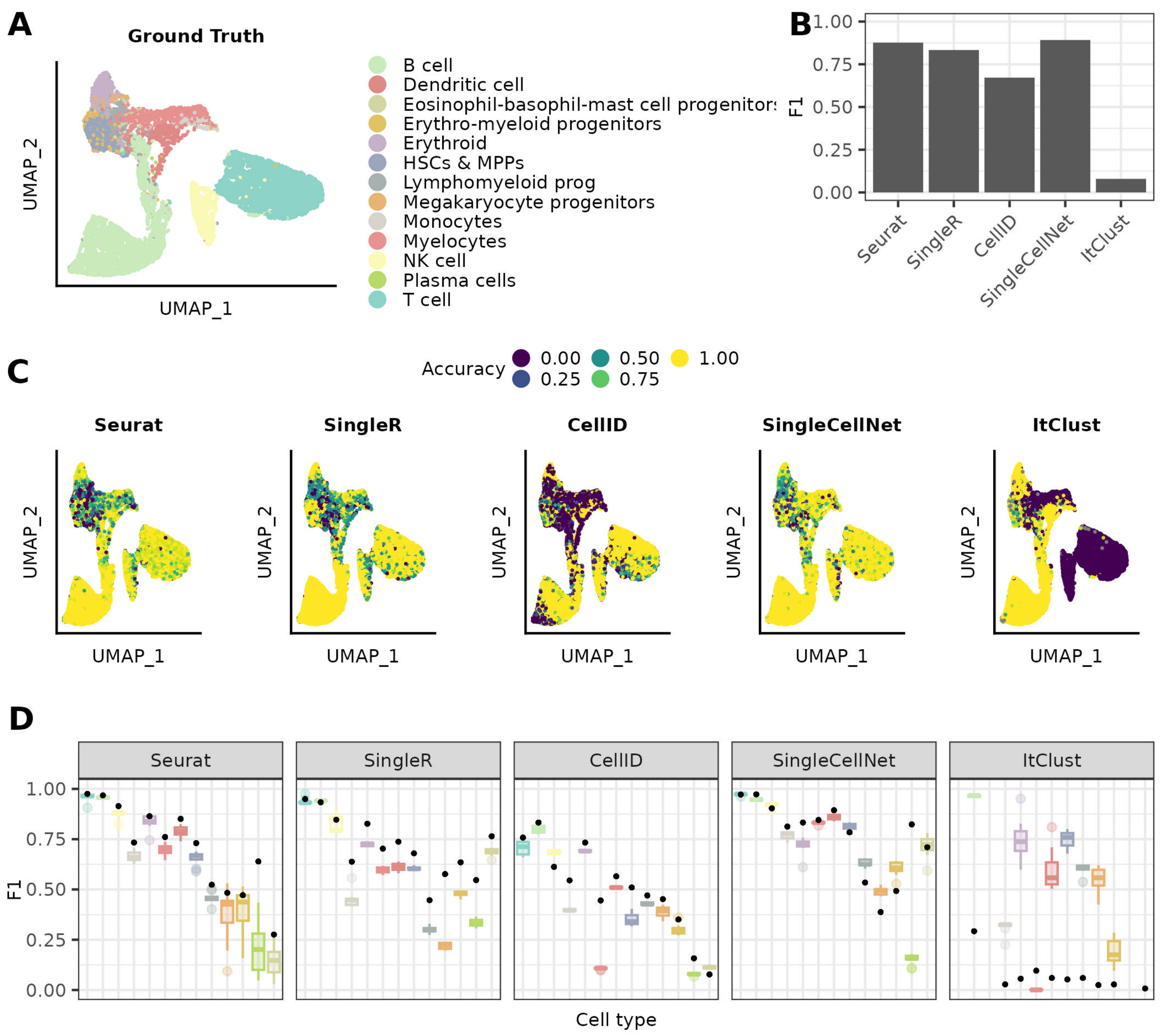


**Figure S8. Cell type label transfer on the human bone marrow dataset.** (A) UMAP colored by the cell type labels assigned in the ground truth. (B) F1 scores achieved by the individual methods when using the full reference data. (C) UMAPs colored by the accuracy achieved for each cell for the individual transfer methods when using them with weighted bootstrapping. (D) Distribution of the F1 scorers for the individual reference subsets shown as boxplots (see Methods). The black points represents the result achieved on the full reference set.

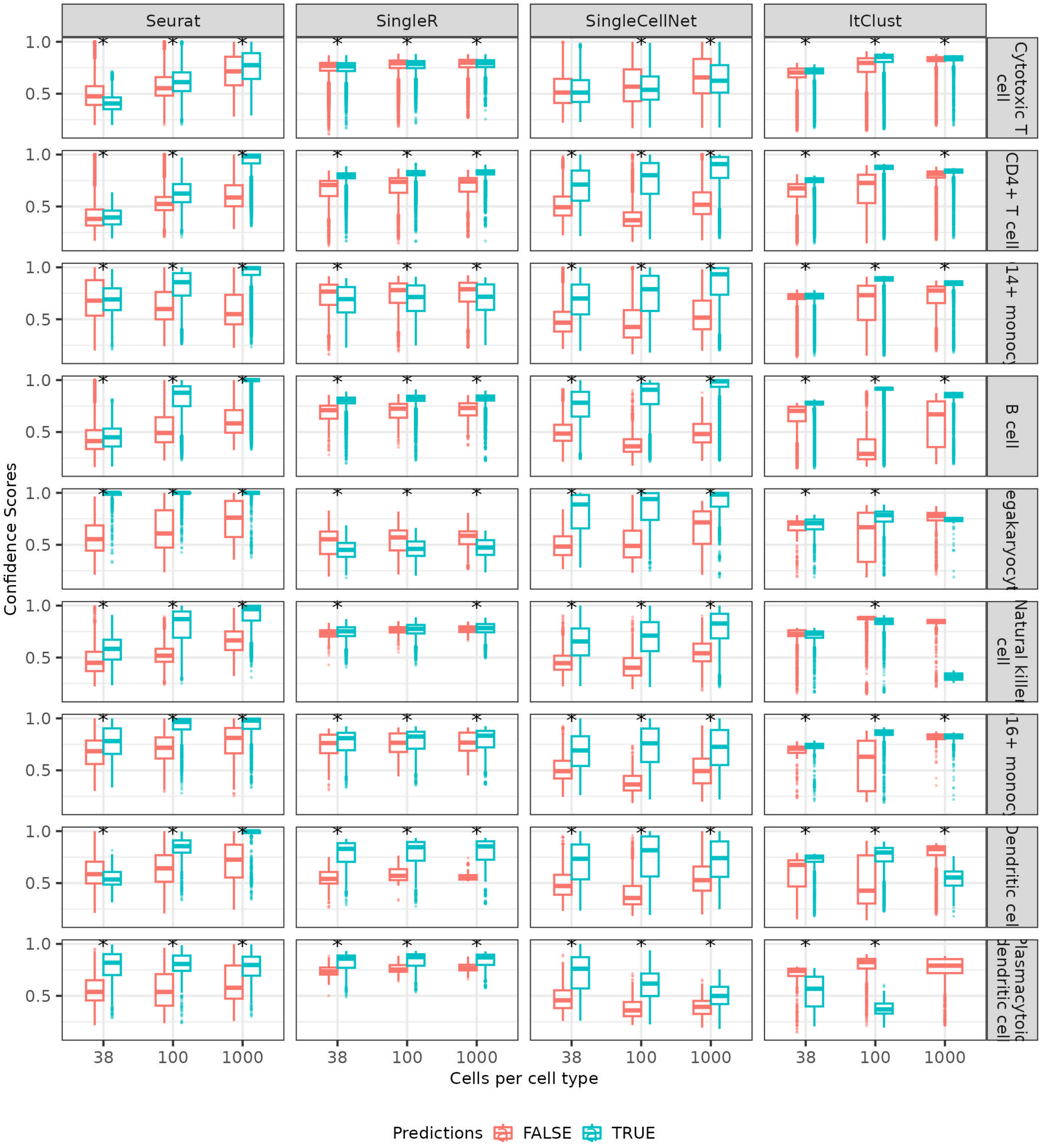


**Figure S9. Distribution of confidence-scores** for the predicted cell types depending of true and false predictions of each of the methods providing confidence scores.
